# Serological screening of SARS-CoV-2 infection in companion animals of Buenos Aires suburbs

**DOI:** 10.3389/fvets.2023.1161820

**Published:** 2023-05-30

**Authors:** Nancy Patricia Cardoso, Carla Rivero, Mariangeles Castillo, Florencia Celeste Mansilla, Florencia Pastorino, Guadalupe Piccirilli, Laura Alonso, Gustavo Martínez, David Di Lullo, Leticia Veronica Bentancor, Alejandra Victoria Capozzo

**Affiliations:** ^1^Instituto de Virología e Innovaciones Tecnológicas “IVIT”, CONICET-INTA, Buenos Aires, Argentina; ^2^Consejo Nacional de Investigaciones Científicas y Técnicas, CONICET, Buenos Aires, Argentina; ^3^Instituto de Estudios para el Desarrollo Productivo y la Innovación, Universidad Nacional de José Clemente Paz, Buenos Aires, Argentina; ^4^Departamento Zoonosis Urbanas, Ministerio de Salud de la Provincia de Buenos Aires, Buenos Aires, Argentina; ^5^Instituto Multidisciplinario de Salud, Tecnología y Desarrollo “IMSaTeD”, CONICET-UNSE, Santiago del Estero, Argentina

**Keywords:** COVID-19 pandemic, multispecies serology, transmission dynamics, companion animal lifestyle, responsible animal care, seroepidemiology studies

## Abstract

The Coronavirus Disease 2019 (COVID-19) is a zoonotic disease caused by the pandemic virus SARS-CoV-2. Domestic and wild animals are susceptible to infection and are potential reservoirs for virus variants. To date, there is no information about the exposure of companion animals in Buenos Aires Suburbs, the area with the largest population in Argentina where the highest number of COVID-19 human cases occurred during the first infection wave. Here we developed a multi-species indirect ELISA to measure antibodies reactive to the SARS-CoV-2 receptor-binding domain (RBD) from several vertebrates constituting the class Mammalia, making it a valuable tool for field serosurveillance. The ELISA cut-off value was estimated by sera from dogs, cats, cattle, and pigs sampled before 2019 (*n* = 170), considering a 98% percentile and a grey zone to completely exclude any false positive result. Specificity was confirmed by measuring levels of neutralizing antibodies against canine coronavirus, the avidity of specific antibodies, and their capacity to impede the binding of a recombinant RBD protein to VERO cells in an In-Cell ELISA. Sera from 464 cats and dogs sampled in 2020 and 2021 (“pandemic” samples) were assessed using the RBD-ELISA. Information on COVID-19 disease in the household and the animals’ lifestyles was collected. In Buenos Aires Suburbs cats were infected at a higher proportion than dogs, seroprevalence was 7.1 and 1.68%, respectively. Confirmed COVID-19 in the caregivers and outdoor lifestyle were statistically associated with seropositivity in cats. The risk of cats getting infected living indoors in COVID-19-negative households was null. The susceptibility of mammals to SARS-CoV-2, the possibility of transmission between animals themselves and humans, together with the free-roaming lifestyle typical of Buenos Aires suburban companion animals, urge pursuing responsible animal care and avoiding human interaction with animals during the disease course. The multi-species RBD-ELISA we developed can be used as a tool for serosurveillance of SARS-CoV-2 infection in mammalians (domestic and wild), guiding further targeted virological analyses to encounter susceptible species, interspecies transmission, and potential virus reservoirs in our region.

## 1. Introduction

Coronavirus disease 2019 (COVID-19) is a highly contagious infectious disease caused by the pandemic severe acute respiratory syndrome coronavirus 2 (SARS-CoV-2) (1). SARS-CoV-2 is a zoonotic pathogen (2) that can infect numerous animal species. In addition to human angiotensin-converting enzyme 2 (ACE2), the Spike glycoprotein of SARS-CoV-2 has a broad host tropism for mammalian ACE2 receptors (3). A recent report accounts for both human-to-cat and cat-to-human SARS-CoV-2 transmission (4). The most striking impact of COVID-19 in animals were the outbreaks at the mink farms in the Netherlands, Denmark, United States, Italy, and Spain (2, 5, 6) where viral infection was probably introduced by infected farm workers to minks (7) and then minks transmitted the virus back to humans (8). The confirmed virus circulation in animal species and the potential development of animal reservoirs impacts on SARS-CoV-2 evolution and pose a risk on public health (9, 10).

Dogs ACE2 receptors are similar to human (11). Freuling et al. showed the susceptibility of raccoon dogs to SARS-CoV-2 infection following intranasal inoculation (12). Virus shedding was detected in nasal and oropharyngeal swabs of infected dogs two days after infection, and these animals transmitted the virus to contact animals. Human caretakers-to-dog transmission has been documented, confirmed by sequencing the infecting virus (13).

Cats are more susceptible to SARS-CoV-2 than dogs (14). Several studies revealed that the virus replicates only in the upper respiratory tract of cats and this replication was not associated with severe disease or death, except in kittens or cats with comorbidities (15, 16). Cats can transmit the infection to other cats (17). Airborne transmission of SARS-CoV-2 between experimentally infected cats implied the feasibility of cat-to-human transmission, which was demonstrated recently (4). It can be argued that the role of pet-cats in transmitting the disease is limited because they are indoor animals (18), while dogs are always considered more actively immersed in human daily life with closer interaction with other people apart from their own household members. However, this may not be the case in many suburban areas in countries like Argentina, where cats are not exclusively indoor animals and they circulate freely between houses and wander in the street, making contact mostly with other cats and in a lesser extent, with other humans than their caregivers. There are also feral cats that interact with cared companion animals, sharing food and water bowls, where the dynamics of companion animals’ interaction with humans is far from having a “safe” pattern. In fact, in these countries, cats and dogs are usually introduced into the household from the streets, and they do not usually receive veterinary care or routine vaccination. This scenario poses a distinct epidemiological context for SARS-CoV-2 transmission compared to that of suburban areas in developed countries.

The unchecked transmission of SARS-CoV-2 in animal hosts could lead to virus adaptation and the emergence of novel variants. Detection of anti-SARS-CoV-2 antibodies is the best way to monitor viral circulation, as viral shedding is usually feeble and brief in cats and dogs. SARS-CoV-2 RBD used as an ELISA antigen is more accurate than nucleoprotein (N)-based ELISA in monitoring for anti-SARS-CoV-2 antibodies (19). Driven by the One Health approach, we established integrated laboratories that use a multi-species and multi-antibody serological kit in ELISA format, which can evaluate the presence of total specific antibodies against SARS-CoV-2 receptor binding domain (RBD), both from animals and humans. This ELISA was initially validated with human samples and approved for human use by the Argentinean Regulatory Authority for human medical products (ANMAT) named “SEROCOVID Federal.” This study had two objectives; one was to verify the performance of our RBD-ELISA with animal serum samples and secondary, to pursue a prospective study to estimate seroprevalence in cats and dogs in Buenos Aires Suburbs during 2020 and analyze the situations that can pose a risk of infection to these animals.

## 2. Materials and methods

### 2.1. Serum samples

Our study enrolled 634 serum samples from animals collected before and during the COVID-19 pandemic and gently provided by the “Departamento de Zoonosis Urbana” Ministry of Health of Buenos Aires Province, Veterinary Hospital of Avellaneda, Buenos Aires; the University of Buenos Aires; the “Instituto Multidisciplinario de Salud, Tecnología y Desarrollo,” CONICET – National University of Santiago del Estero, Argentina and the National Institute of Agricultural Technology INTA. The “pre-pandemic” group comprised 170 frozen serum samples collected between 2016 and 2018 from 115 dogs, 35 cats, 10 cows, and 10 pigs. The other group consisted of “pandemic” samples including 204 dogs, 251 cats, and 9 non-specified companion animals. Samples were stored at-20°C until used.

Many pre-pandemic samples were from dogs undergoing or recovered from other viral. Previous vaccination history or data on viral infections were given. The hospital also provided detailed information on clinical symptoms (22 dogs and 8 cats) among pandemic samples, mainly fever, gastrointestinal and respiratory symptoms. Some animals had been diagnosed as infected by SARS-CoV-2 by RT-PCR and a few of them have been sampled at least once after the diagnosis. We could not gather information on household COVID-19 cases after the animal was diagnosed, as usually the animals were controlled after their caretakers’ convalescence. There were also samples from cats or cats and dogs found on their own on the streets, a common scenario in Buenos Aires suburbs. The analysis of the total pandemic samples also includes cats and dogs from other cities in Argentina, where animals are in closer contact with wildlife. A complete list of samples and the collected information is provided in Supplementary File 1.

Positive and negative controls from SEROCOVID FEDERAL (Laboratorios Chaqueños SA, Chaco, Argentina) were also used, together with a rabbit polyclonal antibody to SARS-CoV-2 RBD (Antibodies.com, catalogue A121533) included during the set-up of the assay. Samples used for setting up the assay’s conditions are shown in Table 1.

**Table 1:** Samples used for RBD-ELISA set-up.

SAMPLE	DAY 1	DAY 2	DAY 3
**BLANK**	0.0402	0.0399	0.0379
*No serum added*	0.0444	0.0382	0.0438
**NEG CONTROL (KIT)**	0.0567	0.0642	0.0657
*Control SEROCOVID*	0.0565	0.0603	0.0688
**POS CONTROL (KIT)**	1.1299	1.1599	1.1841
*Control SEROCOVID*	1.1396	1.1574	1.2175
**RT-PCR+ CANINE**	1.2946	1.2352	1.2782
*Animal diagnosed with COVID-19*	1.2878	1.2797	1.2544
**COMMERCIAL SERUM**	0.8964	0.9157	0.8957
*Rabbit anti-RBD*	0.8866	0.8985	0.9085

Mean values of triplicates are indicated. Samples were run in three consecutive days by different operators.

Serum samples from animals of different species were also used to analyze the reactivity of the detector antibody. The complete list of species analyzed is shown in Table 2.

**Table 2:** Conjugate reactivity assessment.

*Species*	Reactivity	Number of samples
*Bos taurus*	++	10
*Canis familiaris*	++	10
*Chrysocyon brachyurus*	++	4
*Equus ferus*	++	4
*Feliz catus*	++	10
*Homo sapiens*	++	10
*Hydrochoerus hydrochaeris*	++	2
*Leopardus pardalis*	++	1
*Lycalopex griseus*	++	1
*Lynx rufus*	++	3
*Myotis Nigricans*	++	4
*Myrmecopha gatridactyla*	++	1
*Nasua nasua*	++	2
*Oryctolagus cuniculus*	++	1
*Panthera leo*	++	2
*Panthera tigris*	++	4
*Puma concolor*	++	4
*Sus scrofa*	++	10
*Tadarida Brasiliensis*	++	10
*Tamandua tetradactyla*	++	1
*Mus musculus*	+	10
*Desmosdus Rotundus*	+	10
*Didelphis albiventris*	−	1
*Jabiru mycteria*	−	1
*Buteo fuscescens*	−	1
*Ara militaris*	−	1
*Tyto alba*	−	2
*Chelonoidis chilensis*	−	1
*Salmo salar*	−	5
*Psittacoidea sp*	−	5
*Columba livia*	−	5
*Ramphastos sulfuratus*	−	1
*Gallus gallus*	−	4

Performance of the kit’s conjugate with antibodies from different animals of species. Comparable reactivity rates are indicated by greyscale. ++, OD values 450 nm ≥ 1 (comparable to human sera); +, OD values between 0.70 and 0.99.

### 2.2. ELISA procedures

A commercial kit was used (SEROCOVID Federal, Laboratorios Chaqueños SA). This kit was developed by our group at INTA and UNPAZ and transferred to Laboratorios Chaqueños SA. The protocol we used followed the manufacturer’s recommendation. Briefly, serum samples were diluted 1:50 in Dilution Buffer, added to each well of RBD (20) pre-coated plates provided in the kit, and incubated for 30 min at 37°C. After four washing steps with Washing Solution the presence of RBD-specific antibodies was revealed using a 1:10,000 dilution of a horseradish peroxidase (HRP)-conjugate (trade secret) followed by a 30 min incubation at 37°C and the subsequent substrate addition of TMB (TMB X-tnd, Kementec, Denmark) after washing (4 times). The reaction was stopped with 0.16 N H2SO4, and plates were read at 450 nm using a microplate reader (Infinite F50, Tecan). OD values of the samples and controls were corrected by subtracting mean blank OD values and analyzed without further calculations.

The avidity of RBD-specific antibodies was determined by incubating plates after the serum samples binding step with 200 μL per well of 6 M urea in PBS for 20 min after the first PBST washing step. The ELISA then followed the protocol previously described. The percentage of residual reactivity due to the urea-wash treatment was calculated and expressed as avidity index “AI% (21).

To analyze the reactivity of the detector antibody (multi-species HRP conjugate, trade secret), microplates (Greiner Bio-One™ crystal clear polystyrene high binding) were coated with each serum sample from different species diluted 1:5 in carbonate–bicarbonate buffer (pH 9.6) as capture antigen. After an ON incubation at 4°C, plates were blocked with 30% skim milk-PBST (250 μL/well) and subsequently incubated for 15 min at room temperature. Following two washing steps, the reactivity was evaluated using a 1:10.000 dilution of the kit’s HRP-conjugate followed by 30 min of incubation at 37°C and the subsequent TMB substrate addition after the washing. The reaction was stopped with 0.16 N H2SO4, and plates were read at 450 nm (Infinite F50, Tecan).

### 2.3. RBD-blocking in-cell ELISA

The capacity of the anti-SARS-CoV-2 antibodies present in serum samples to block the binding of the RBD to its receptor (ACE2), was assessed by an in-cell ELISA. Briefly, VERO cells were seeded on 96-well plates (GBO MicrolonTM, Thermo Fisher, DE, United States). Once the confluence reached 90%, the plate was washed with PBST 0.05% (washing buffer), fixed with PBS-PFA 4% (100 μL/well; 20 min) and blocked with blocking buffer (PBS-10% fetal calf serum) for 90 min. Both incubations were performed at room temperature. Meanwhile, sera diluted 1:50 in blocking buffer were incubated with a recombinant RBD (10 μg/mL; 50 μL/well) for 30 min at 37°C. The purified recombinant RBD expressed in *P. pastoris* was gently provided by “Facultad de Ciencias Exactas y Naturales” of the University of Buenos Aires (UBA) through the Argentinean anti-COVID Consortium (20). Then serum-RBD mixtures were transferred to the culture plate and incubated for 1 h at 37°C. After three PBST washing cycles, a rabbit anti-RBD polyclonal antibody (50 μL/well, 1:500, Antibodies.com, United Kingdom) was added and incubated for 1 h at 37°C, followed by an anti-rabbit-HRP conjugate (50 μL/well, 1:10.000, ThermoFisher). The reaction was revealed with ABTS-H2O2 (50 μL/well; Sigma, San Luis, MO), stopped with a 1 M NaF solution, and read at 405 nm. To normalize cell density between wells, Janus Green staining was applied (Sigma) as previously described (22).

### 2.4. Canine coronavirus neutralization test

The presence of serum-neutralizing antibodies anti-canine Coronavirus (cCoV) was assessed using a standard variable serum-fixed virus method. Briefly, serial two-fold dilutions of each serum sample (starting at 1:15) were incubated on 96-well culture plates with 300 DICT50 of infective culture-adapted cCoV for 1 h at 37°C 5% CO2. Then 100 μL of a suspension containing 2.5 × 105 CRFK (Crandell-Rees Feline Kidney) cells were added to the serum-virus mixture, and plates were incubated at 37°C, 5%CO2 for 72 h. The neutralizing titer of the analyzed samples was determined according to the number of protected replicates in the serial dilutions based on the Reed and Muench interpolation method (23), expressed as the logarithm (base 10) of the reciprocal of the last dilution of serum that neutralized 100 × TCID50 of the virus in 50% of the wells.

### 2.5. Statistical analysis

The 1-way or 2-way ANOVA was used as long as the data followed a normal distribution (Wilk–Shapiro Normality Test) and their variances were comparable (Bartlett’s Test). Analyses were performed using 2-way repeated measures ANOVA followed by a Bonferroni multiple comparison test. When the number of data was not sufficient to verify the normal distribution, the analysis of the differences was performed using non-parametric methods: Mann–Whitney for comparisons between two groups or Kruskal–Wallis for multiple comparisons. If significant differences were found in the Kruskal–Wallis test, Dunn’s test was performed for pairwise comparisons.

The association (contingency analysis) between the risk of infection related to the COVID-19 situation in the household or lifestyle was estimated using Fisher Exact Test for 2 × 2 tables.

A minimum confidence interval of 95% was used for all the statistical analyses.

## 3. Results

### 3.1. Set-up of the assay

**Figure 1:**
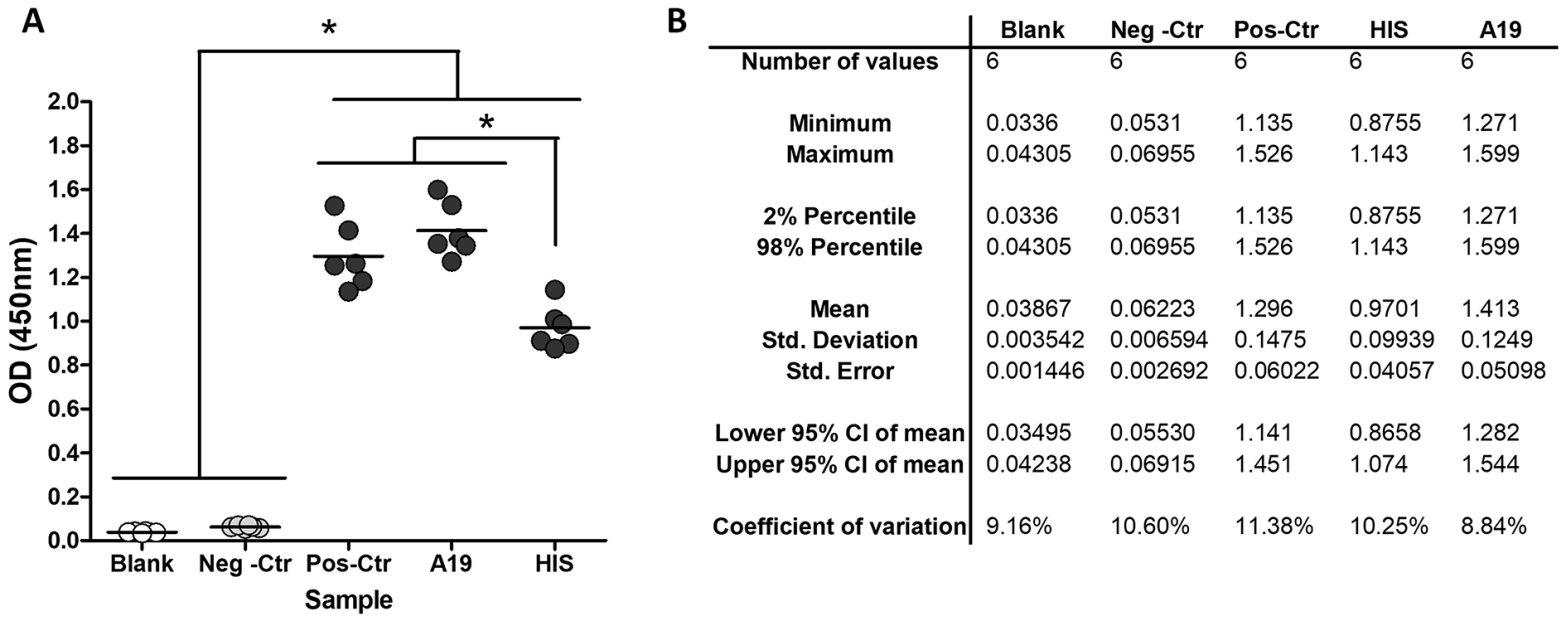
RBD-ELISA performance. **(A)** OD values of blanks and control samples, including a commercial hyperimmune rabbit serum (HIS) and a COVID-19 RT-PCR confirmed dog (A19) sampled 21 days post-infection. **(B)** Inter-plate variation using control samples.*p < 0.05 (Kruskal–Wallis followed by Dunns test).

The assay’s general conditions and steps were set up using the kit’s positive and negative controls (SEROCOVID Federal), a serum sample from an RT-PCR COVID-positive dog (A19) that was sampled 25 days post-COVID diagnosis, and a commercial rabbit serum reactive to RBD. Samples were tested in different dilutions and 1:50 was selected as the most appropriate, yielding high OD values in the positives and values like the blanks, for the negatives (Figure 1). The conjugate was used at the same dilution as the one indicated in the kit and the blocking buffer was modified to ensure a better difference between OD values of positive and negative animal samples. The modified blocking buffer was used to manufacture the plates that were used pre-coated and pre-blocked, for further evaluation. In these conditions, the RBD-ELISA could discriminate between the commercial serum and positive kit control or A19 (Figure 1). Using these samples, we assessed intra-assay reproducibility and inter-assay repeatability. Samples were run in duplicates in six independent ELISA plates for three consecutive days with different operators. Inter-plate variations were between 8 and 11% depending on the sample (Table 1).

The capacity of the conjugate to detect antibodies from different species was tested by directly binding the sera to the ELISA plate diluted 1:50 in the coating buffer. Most mammal samples yielded OD values comparable to human samples, considering a 15% variation (Table 2). Those detected as positive but with values between 15 and 25% below human ODs are indicated with one plus sign (Table 2). For these samples, a 1:25 instead of 1:50 increased the detection capacity of the antibodies. The preliminary analyses we did by testing various species show that the kit can detect antibodies from individuals of the class Mammalia, while it cannot be used for reptilians, amphibians, or birds.

### 3.2. Performance of the RBD-ELISA with pre-pandemic samples from companion and production animals

**Figure 2:**
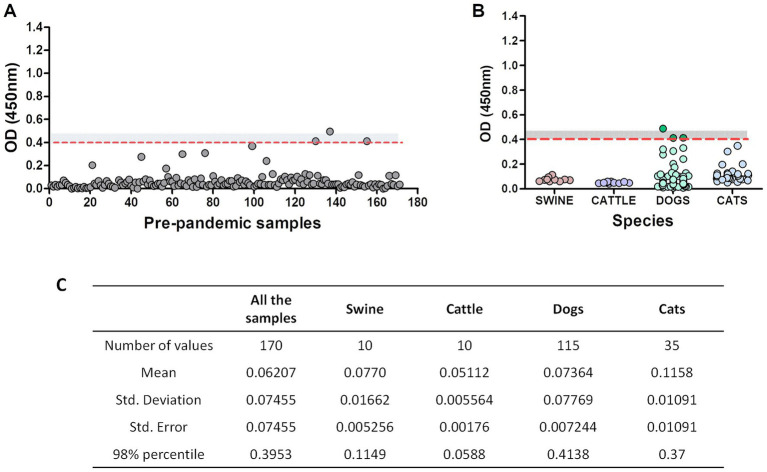
Pre-pandemic serum samples assessment using RBD-ELISA. OD values of the 170 pre-pandemic samples altogether **(A)** or discriminated by species. **(B)** The dotted red line depicts the 98% percentile cut-off value. The grey zone (undetermined results) is also shown as a grey-dotted area (values between OD = 0.4 and OD = 0.45). Samples with an OD < 0.4 are considered non-reactive and informed as negative, meaning absence of antibodies reactive to RBD. Samples with an OD > 0.45 are considered reactive (positive). **(C)** Statistical analysis of the samples.

Serum samples from companion animals collected before 2018 “pre-pandemic samples” (*n* = 170) were analyzed using the RDB-ELISA. The mean OD for these samples was 0.06, very similar to the blank’s OD values (OD ~ 0.04). Using the 98% percentile, the cut-off value was OD = 0.40 (Figure 2A). By adding a “grey zone” up to OD = 0.45, specificity increased up to 99.2%. Only one animal fell above this threshold, while the other two samples fell within the grey zone (OD values: 0.414 and 0.413). Samples from cats, cattle and pigs did not surpass the OD = 0.4 cut-off value (Figure 2B). The 98% percentile-OD threshold was different between the tested species, being higher in dogs (0.41), followed by cats (0.37), swine (0.115), and cattle (0.058; Figure 2C).

We then focused on the three “outlier” dogs (depicted in dark green, Figure 2B). These animals were semi-feral, they approached the dairy farms to eat the placenta and stillborn calves and they never received veterinary care. Surprisingly, suburban animals living in human households yielded much lower OD values (Figure 2B, light green dots). We then considered the possible assay interferences, like the presence of low-avidity antibodies due to cross-reactivity with other viruses (such as canine coronavirus), or the lack of biological activity of the antibodies detected by the kit. To further explore these alternatives, we assessed the avidity of the anti-RBD antibodies by including a urea-washing step of the serum sample, titrated anti-canine coronavirus neutralizing antibodies and verified the capacity of these antibodies to prevent the binding of RBD on VERO cells using an in-cell blocking ELISA. For comparison purposes, pre-pandemic samples and post-pandemic randomly selected negative and positive samples were included in the same plate. Results are summarized in Table 3.

**Table 3:** Studies on the RBD-specificity of three outlier canine pre-pandemic serum samples.

Sample	RBD ELISA (OD 450 nm)	AVIDITY ELISA (OD 450 nm)	AI (%)	RBD binding inhibition (%)	VNT (cCoV)
PreP-DOG	POS (0.4970)	0,28	47	90	30
PreP-DOG	GREY (0.413)	0,34	82	94	Neg
PreP-DOG	GREY (0.414)	0,19	46	71	Neg
PreP-DOG	NEG (0.3704)	0,13	35	35	20
Pand-DOG	NEG (0.27)	0,13	ND	33	30
Pand-DOG	NEG (0.27)	ND	ND	42	500
Pand-DOG	POS (0.46)	0,02	5	50	100

Pre-pandemic and pandemic dog samples (PreP and Pand, respectively) were analyzed by the RBD-ELISA and the residual reactivity assessed by avidity ELISA (Avidity index “AI”). RBD binding inhibition in by each sample is also shown, as well as Canine coronavirus (cCoV) neutralizing titers (Dilution^−1^).

The pre-pandemic dog with low-positive serology (OD = 0.497) yielded low avidity anti-RBD antibodies and low canine coronavirus VNT titers. This serum almost completely inhibited the binding of RBD to VERO cells. Dogs’ samples with OD values within the grey zone also inhibited RBD binding. Neutralizing antibodies against canine coronavirus were negative. RBD-ELISA negative pandemic dog samples did not inhibit RBD binding to VERO cells. One of these animals had high anti-canine coronavirus VNT titers. The seropositive pandemic sample had low-avidity antibodies that were only partially capable of inhibiting RBD binding to VERO cells (Table 3). Avidity indexes were positively related to the capacity of the serum to inhibit RBD binding to VERO cells.

### 3.3. Serology in companion animals: pandemic samples

**Figure 3:**
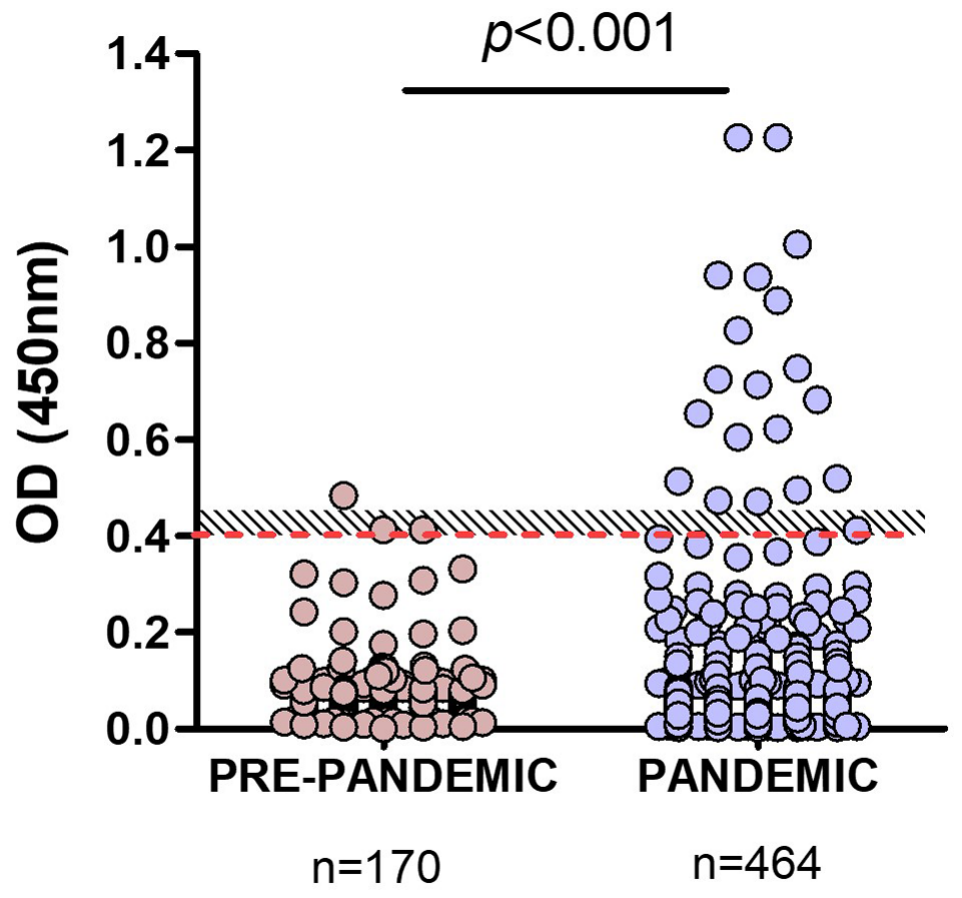
Comparison of RBD-ELISA outcome using animal pre-pandemic and pandemic serum samples. The red-dotted line depicts the OD = 0.4 cut-off value and the shaded area, the “grey zone” (unclassified samples). Significant differences (*p* < 0.001; Mann–Whitney test) between OD values of samples taken before or during the pandemic are indicated.

Pandemic samples from companion animals (*n* = 464) were then tested with our RBD-ELISA and OD values compared to those of pre-pandemic samples. The assay detected significant differences (*p* < 0.001) between OD values of samples taken before or during the pandemic (Figure 3). Among pandemic samples, we found a 4% overall positivity considering values above OD = 0.45 (above the grey zone), and 4.96% using the OD = 0.4 cut-off value.

**Figure 4:**
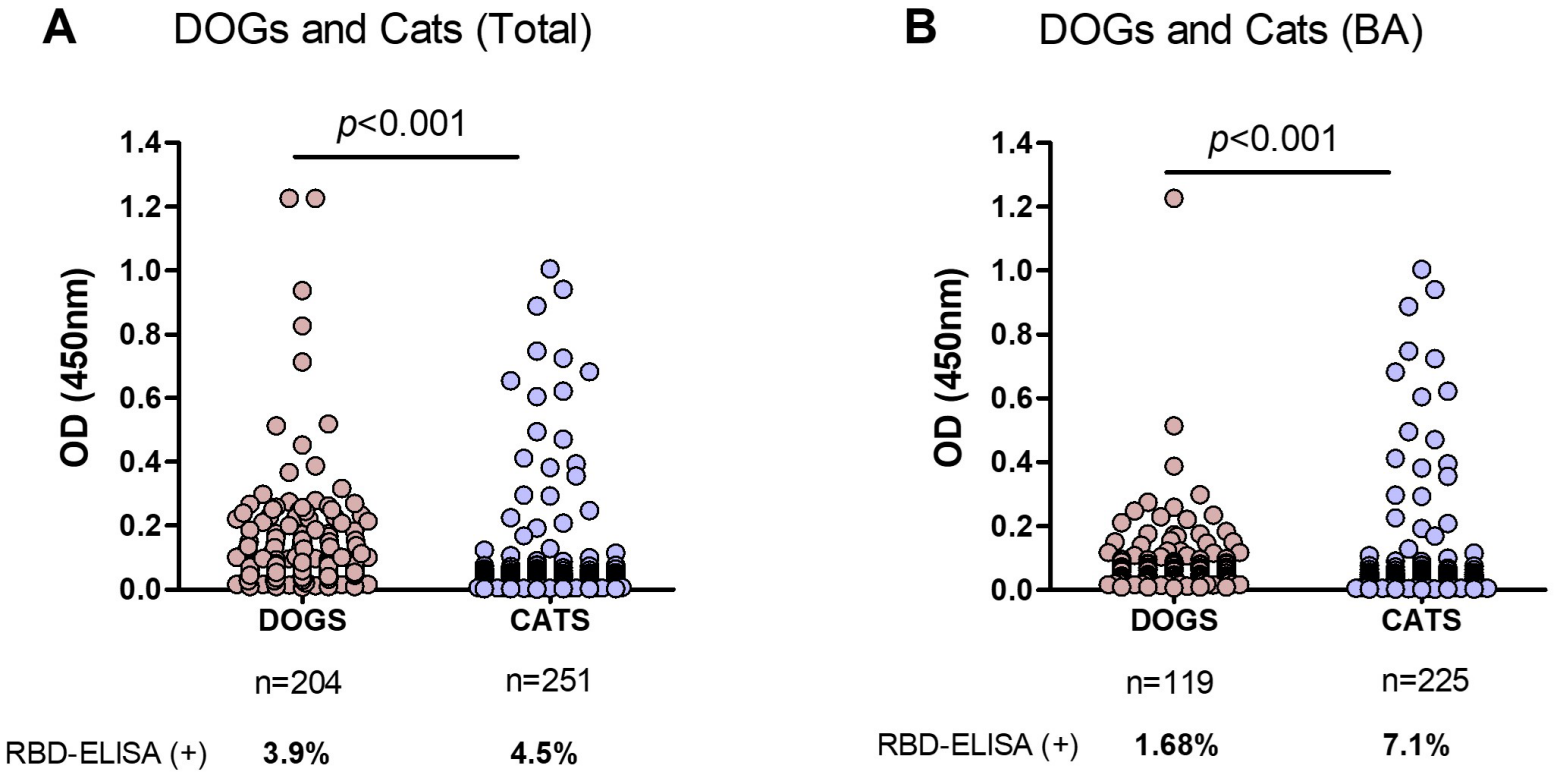
Comparison of RBD-ELISA outcome in companion animals’ serum samples. **(A)** OD results from dogs and cats from Buenos Aires and other provinces, excluding nonidentified species and **(B)** Dogs and cats from Buenos Aires suburbs (BA). The number of samples and percentage of animals with positive serology are indicated below each graph. Significant differences between dogs and cat’s OD values are indicated (Mann–Whitney test).

Samples from dogs and cats were analyzed, excluding those of unknown companion-animal species. In the first analysis, we included samples from Buenos Aires and other different provinces (Figure 4A). Using the OD = 0.4 cut-off value seropositivity was 4.39 and 3.96% considering the grey zone. The percentages of positive samples, meaning previous SARS-CoV-2 infection, were quite similar between dogs and cats: 3.9 and 4.5%, respectively.

Interestingly, 78% of the COVID-19 serology-positive dogs belonged to households with confirmed COVID-19 disease. Three serology-positive dogs had been sampled after their caretakers´ RT-PCR confirmed COVID-19 diagnosis. Many of these animals developed gastrointestinal symptoms. One of these animals with positive serology (OD = 1.217) suffered from a long-term gastrointestinal disease, decay, fever, and loss of smell.

In a second analysis, we only considered samples from Buenos Aires Suburbs (Figure 4B), an area with the highest COVID-19 positive rate in the country. In this case, the overall positivity was 6.4 and 4.5% when considering values above OD = 0.4 or 0.45, respectively. In this subgroup, positive serology was significantly higher in cats compared to dogs (7.1 and 1.7%, respectively; *p* < 0.001), indicating that in an active viral circulation environment, cats became infected at higher rates than dogs.

**Figure 5:**
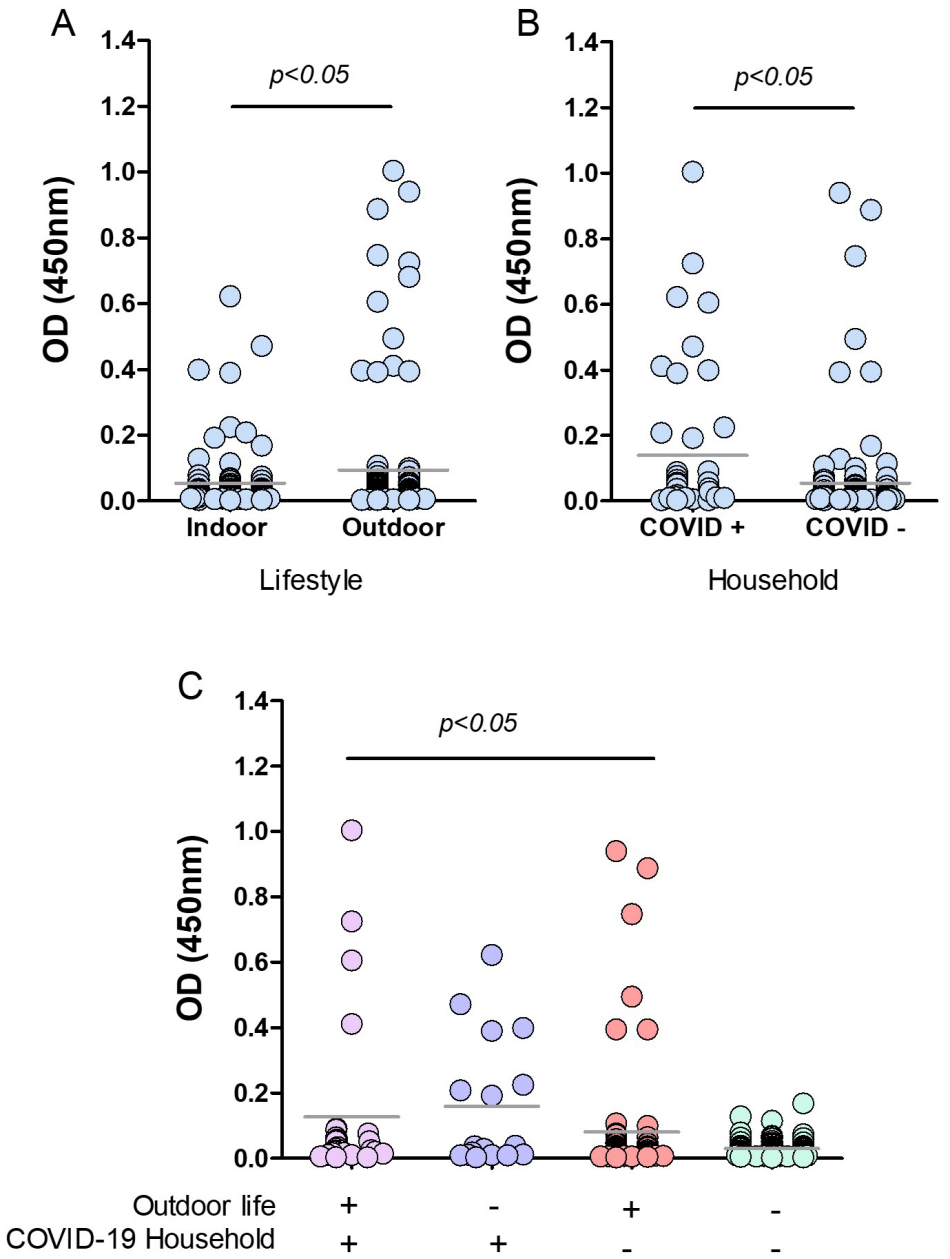
Risk factors in cats from Buenos Aires suburbs. RBD-ELISA results were grouped according to the cats’ lifestyle **(A)**, the presence or absence of COVID-19 human cases in their homes, **(B)** or all the possible combinations of both parameters, and **(C)** Grey horizontal lines depict mean values. Feral animals were excluded from analysis in graphs B and C. Significant differences are indicated: Mann–Whitney test in graphs A and B; Kruskal–Wallis followed by Dunns in graph C.

Information on the cat’s habits, lifestyle, and cohabitation with humans with or without confirmed SARS-CoV-2 infection was provided, except for 22 animals. We grouped cats` OD values in those with reported symptoms, exclusively indoor cats (those that never left the house) and outdoor animals, including feral cats and those having mixed indoor-outdoor behavior: living in a home but roaming around by their daily (Figure 5A). We only had data from 8 cats with symptoms at the time of sampling; none of them had antibodies reactive to RBD (data not shown).

Cats with outdoor life yielded mean anti-RBD antibody values that were significantly higher than those of indoor cats (*p* < 0.05; Figure 5A). Only 4.4% of the indoor cats yielded positive serology compared to 10.7% (12 out of 112) in outdoor animals. Among outdoor animals, there were 14 feral cats; two of them had positive serology, which implies a 14% of positive serology in this subgroup. We then analyzed the data by categorizing animals according to the COVID-19 situation in their households. Feral animals were excluded from this analysis. The data show that cats co-living with SARS-CoV-2 infected humans have higher mean OD values than those living in COVID-negative homes (*p* < 0.05; Figure 5B). The percentage of seropositive animals was 18% (8 out of 45) and 4.2% (6 out of 142) for COVID-19-positive and negative households, respectively.

Analysis of OD values comparing and combining the different conditions in lifestyle and contact with infected humans show that antibody levels in cats with outdoor habits (either living or not in COVID-19 households) and those indoor animals in contact with infected caregivers were significantly higher than antibody levels of indoor cats living in non-COVID-19 homes (*p* < 0.05; Figure 5C). Among outdoor-lifestyle home-cared cats, only 30 belonged to COVID-19 households, and 4 of them were infected (13%). The rest lived in COVID-19-negative homes, and positive serology in this group was 3.9%, meaning they got infected by individuals outside their homes. It also means they did not transmit the virus to their caregivers unless they all underwent asymptomatic infections.

The absence of COVID-19 infection in these cats (negative serology) was statistically associated with living indoors in a non-COVID-19 household. In this condition, the risk of infection was estimated as null (predictive value confidence interval 0.91**–**1; Fisher’s exact test *p* value = 0.0112). Compared to those animals living indoors in a COVID-19-negative household, we found significantly higher anti-RBD antibody levels in indoor cats co-living with infected humans (23.5%; *p* < 0.05) and in outdoor stray wandering cats, living in COVID-19-positive households (13.3%).

The lifestyle was also associated with a differential risk of infection (*p* = 0.044, Fisher’s exact test). The serology negative predicted value was 0.95, meaning that indoor habit is a low-risk situation for SARS-CoV-2 transmission.

## 4. Discussion

COVID-19 is a zoonosis and several animal species are susceptible and in risk of becoming SARS-CoV-2 reservoirs (24). One example was the infection of minks as a source of viral diversity that contributed to the growth of the human pandemic (8, 24, 25). Other animals, including domestic animals, have also been associated with COVID-19 infection (24). In this scenario, the use of serological tests that can detect antibodies in susceptible species to guide virus surveillance is paramount. The indirect RBD-ELISA kit we developed was approved by the Argentinean regulatory agency (ANMAT) as a multi-species kit for human use in March 2021. In this study we verified the kit’s conjugate reactivity with antibodies from several species of the class Mammalia which are among those considered susceptible to SARS-CoV-2 infection, with a potential role in transmission (24). The conjugate cannot react with antibodies from fish, birds, or reptiles. Recent studies suggested that SARS-CoV-2 can potentially bind to the ACE2 proteins of a broad range of mammals as ACE2 genes of several mammalian species were found to be highly conserved (24), while for example, chickens, and ducks were found not to be susceptible (16).

Our RBD-ELISA could differentiate positive from negative samples. Pre-pandemic samples yielded low OD values: 75% were below OD = 0.07 (similar to the blanks OD value). A cut-off was estimated using the 98% percentile of the OD values distribution. Using this cut-off only three canine samples were misclassified. With the addition of a grey zone, we ended up with just one outlier.

We focused on those three outlier dog samples to verify the assay’s specificity. These samples had high avidity anti-RBD antibodies capable of blocking the binding of RBD to the SARS-CoV-2 susceptible cells, meaning the antibodies were specific. They were negative or had low levels of neutralizing antibodies against canine coronavirus. Interestingly, these three dogs were semi-feral animals, that interacted with dairy cattle (especially eating remnants of abortion or birth tissues), wildlife and farm workers. Semi-feral dogs are always found in Argentinean livestock farms, so they should be monitored as potential important actors in COVID-19 epidemiology as vehicles of the virus from domestic to wild settings or *vice-versa*. Feral, semi-feral dogs and foxes are already important in the epidemiology of other diseases. For instance, these three animals were sampled in 2016 as part of a Neosporosis epidemiology study (27).

It has been proposed that RBD or Spike-based ELISAs are more accurate than those using N-antigen due to lower cross-reactivity with other coronaviruses (19). The presence of only three outlier samples out of 170 (1.76%) can be considered a fortuity of the serology. Considering only dogs the percentage of miss-classified samples is 2.6%. Interestingly, OD values from all the pre-pandemic samples from suburban animals under human care did not surpass the cut-off value, while the three outlier samples belonged to the semi-feral dairy farm-visiting dogs. These animals are in contact with wildlife, domestic animals, production animals and humans. It would be interesting to analyze larger numbers of pre-pandemic stored samples to give us a clue about similar coronaviruses already circulating in wild animals in our region before 2020. Another readout is the possible presence of cross-reactive antibodies that might be difficult to rule out, particularly in wild animals, even though a highly specific RBD-based ELISA is used.

RBD-ELISA negative animals did not block RBD binding to SARS-CoV-2 susceptible cells, and had high canine coronavirus neutralizing titers, while a positive pandemic dog with low but positive OD value, had low avidity antibodies concordant with a low capacity to block RBD in the In-Cell ELISA. These results, and other recent data from our laboratory suggest there might be a correlation between the avidity indexes of the sera and their capacity to block RBD binding to ACE2. Further studies are needed to confirm or dismiss this hypothesis.

Serological analysis of pandemic samples revealed an overall 4% positivity in companion animals, meaning that cats and dogs became infected at lower rates compared to humans. Symptoms have only been identified in serology-positive dogs, which mostly refer to gastrointestinal complications. A recent report by Padilla-Blanco et al. suggests that SARS-CoV-2 could affect the gastrointestinal tract of dogs (28). Most of the serology-positive dogs referred to COVID-19-related symptoms, while this was not evident for cats. It is worth noting, however, that 70% of the Buenos Aires suburban serum samples from cats were obtained as part of a program aimed to survey for Sporotrichosis, a highly prevalent mycosis in our region, so information on symptoms was not provided for these animals.

If we analyze our results by discriminating between cats and dogs, positive serology was almost equally frequent between them if samples from different parts of the country were analyzed. However, considering only companion animals living in Buenos Aires suburbs, cats showed a higher frequency of positive serology than dogs (Figure 4). The Buenos Aires suburban area was the region with the highest human COVID-19 prevalence in the country, and it is also the area with the highest population density. It is then expected to have elevated infection rates, as SARS-CoV-2 circulated intensively in this area. Similar results have been described in other heavy-populated areas around the world (29–31). This can also account for the similarity and lower infection rates seen between dogs and cats in those areas with lower SARS-CoV-2 circulation rates.

Only a few RT-PCR-positive animals were sampled after their diagnostic, and their sera yielded positive in our RBD ELISA. Most of the caretakers assisted the clinic with a consultation about different symptoms, or to verify if their companion animals had been infected after their convalescence, so we could not identify any animal infection before the onset of the disease in the household. However, animal-to-human transmission is possible, and the presence of companion animals in the house was identified as a risk of infection by SARS-CoV-2 (18). Cases were reported among farm minks’ farmworkers (5, 32) and there was a recent case of cat to human transmission in Thailand (4). Cats usually shed low amounts of virus (15, 19, 33, 34) and given that since the beginning of the pandemic by December 2019, only a few transmission cases were reported until now, the companion animal-to-human transmission route may not the main source of virus circulation. However, apart from the precautions clinical veterinarians should take when dealing with sick animals, the most important potential role of either companion, production or wild animals in the pandemic might be to act as reservoirs of SARS-CoV-2. In this regard, serology can be an important tool for surveillance that can guide further targeted virological inspection.

Two risk factors for SARS-CoV-2 infection in suburban cats of Buenos Aires were identified: the presence of human COVID-19 cases within the household and the outdoor lifestyle. The human-to-animal transmission that occurred at a very low rate has been identified as the most likely epidemiological scenario for companion animal infection in other studies (29, 33, 35). An active surveillance study performed in Ecuador showed that transmission from infected owner to household dogs and cats is associated with food sharing (36). Other authors also showed that infection occurred in cats living in a SARS-CoV-2 polluted environment (30). As we said above, Buenos Aires Suburbs was the area with the highest number of human COVID-19 cases in the whole country. Infection of cat populations was first demonstrated in Wuhan during the COVID-19 outbreak based on detection of SARS-CoV-2 specific antibodies in 14.7% of sampled cats (31). In a study performed in France, neutralizing SARS-CoV-2 antibodies were detected in 8.4% of cats (29). In Buenos Aires, we estimated a similar value (10%). In many seroprevalence studies positive serology is usually higher in cats than in dogs, mainly in areas with high case numbers in the human population (29, 30). Considering that suburban cats wander freely between houses and in the street, interacting with other cats, some of them feral and semi-feral cats also visit households and share the same water or food bowls used by the inhabitants’ companion animals, either cats or dogs, animal-to-animal transmission is another possible epidemiological scenario for Buenos Aires suburbs. Serosurveillance of cats found on the streets and their interaction with different regional susceptible species should be pursued.

This study supports the importance of responsible companion-animal ownership to reduce viral infection and posterior transmission or long-term health issues. Another outcome is the importance of using kits like ours that can be applied indistinctly to samples from animals (companion, production and wild) and humans as tools to address the One Health perspective for tackling zoonotic diseases.

In conclusion, our study provided serological evidence for SARS-CoV-2 circulation in companion animals of Buenos Aires Suburbs, being cats more susceptible to infection than dogs. In cats, positive RBD-serology was associated with COVID-19 episodes in the household and with the outdoor social lifestyle of these animals, related to possible animal-to-animal transmission. In addition, we verified that our locally produced kit that can be used with serum samples from mammals to monitor viral circulation to identify animal viral reservoirs and potentially prevent further incursions of SARS-CoV-2 variants in our region.

## Data availability statement

The original contributions presented in the study are included in the article/Supplementary material, further inquiries can be directed to the corresponding author.

## Author contributions

CV and BL conceived and planned the experiments and received the funding for most of the experimental work as PI and co-PI, respectively. CP and RC carried out most of the experiments, with the collaboration of CM. MC set-up and performed In-Cell ELISA and canine coronavirus VNT experiments. PF, PG, AL, and MG contributed to sampling in Buenos Aires suburbs and helped with the epidemiological analysis. DD provided and characterized samples outside Buenos Aires and helped analyze the use of our kit with wildlife samples. CV, BL, CP, DD, and RC contributed to the interpretation of the results. CV took the lead in analyzing the results, preparing the graphs, and writing the manuscript. All authors contributed to the article and approved the submitted version.

## Funding

This study was funded by FONCYT through IP COVID 317 “Desarrollo de Kit serológico multiespecie (ELISA) para la detección de anticuerpos contra el SARS-CoV-2” awarded to LVB (UNPAZ-CONICET) and AVC (INTA-CONICET). Additional funding was received from INTA through the National Animal Health Program (PNSA I105).

## Conflict of interest

The authors declare that the research was conducted in the absence of any commercial or financial relationships that could be construed as a potential conflict of interest.

## Publisher’s note

All claims expressed in this article are solely those of the authors and do not necessarily represent those of their affiliated organizations, or those of the publisher, the editors and the reviewers. Any product that may be evaluated in this article, or claim that may be made by its manufacturer, is not guaranteed or endorsed by the publisher.
